# Clinically important alterations in pharmacogene expression in histologically severe nonalcoholic fatty liver disease

**DOI:** 10.1038/s41467-023-37209-1

**Published:** 2023-03-17

**Authors:** Nicholas R. Powell, Tiebing Liang, Joseph Ipe, Sha Cao, Todd C. Skaar, Zeruesenay Desta, Hui-Rong Qian, Philip J. Ebert, Yu Chen, Melissa K. Thomas, Naga Chalasani

**Affiliations:** 1 Indiana University School of Medicine, Department of Medicine, Division of Clinical Pharmacology, Indianapolis, IN USA; 2 Indiana University School of Medicine, Department of Medicine, Division of Gastroenterology Hepatology, Indianapolis, IN USA; 3 Eli Lilly and Company, Indianapolis, IN USA

**Keywords:** Non-alcoholic fatty liver disease, Translational research, Predictive markers

## Abstract

Polypharmacy is common in patients with nonalcoholic fatty liver disease (NAFLD) and previous reports suggest that NAFLD is associated with altered drug disposition. This study aims to determine if patients with NAFLD are at risk for altered drug response by characterizing changes in hepatic mRNA expression of genes mediating drug disposition (pharmacogenes) across the histological NAFLD severity spectrum. We utilize RNA-seq for 93 liver biopsies with histologically staged NAFLD Activity Score (NAS), fibrosis stage, and steatohepatitis (NASH). We identify 37 significant pharmacogene-NAFLD severity associations including *CYP2C19* downregulation. We chose to validate *CYP2C19* due to its actionability in drug prescribing. Meta-analysis of 16 independent studies demonstrate that *CYP2C19* is significantly downregulated to 46% in NASH, to 58% in high NAS, and to 43% in severe fibrosis. Our data demonstrate the downregulation of *CYP2C19* in NAFLD which supports developing personalized medicine approaches for drugs sensitive to metabolism by the CYP2C19 enzyme.

## Abstract (web summary)

This study characterizes expression of pharmacogenes across the histological NAFLD severity spectrum. Here, the authors show the downregulation of *CYP2C19* in NAFLD which supports developing personalized medicine approaches for drugs sensitive to metabolism by the CYP2C19 enzyme.

## Introduction

Nonalcoholic Fatty Liver Disease (NAFLD) is one of the most common liver diseases, affecting 25% of the world’s population^1^. If untreated, NAFLD progresses to non-alcoholic steatohepatitis (NASH), liver fibrosis, and eventually cirrhosis. While diet, weight-loss, and gastric bypass surgery are effective treatments^2,3^, there are currently no FDA-approved drugs to treat NAFLD. Since NAFLD is highly associated with obesity, diabetes, hypertension, hypercholesterolemia, and cardiovascular disease^4–9^, drugs used to treat cardiometabolic conditions (e.g. clopidogrel) are frequently used in NAFLD patients. However, most of these drugs were not originally studied in NAFLD-specific patient populations. For example, of 3 big trials demonstrating the lifesaving antiplatelet effect of clopidogrel (CURE, CAPRIE, CLARITY-28)^10–12^, none published NAFLD subgroup analyses and one even excluded patients with hepatic insufficiency. Therefore, the approved dosages or other findings from these original drug studies may not be representative of people with NAFLD. Thus, in this study we aimed to determine if NAFLD patients could be at risk of altered drug response by identifying changes in the hepatic expression of genes that mediate drug disposition (pharmacogenes) across histological NAFLD severity.

Pharmacogenes code for proteins involved in the disposition and response to drugs like anti-hypertensives, lipid-lowering agents, anti-platelets, and agents used to treat diabetes. Many of these drugs are substrates for pharmacogenes like the cytochrome P450 (CYP) metabolic enzymes as well as the ATP-Binding Cassette (ABC) and Solute Carrier (SLC) transporters. Changes in the expression of these genes can impact the response to these drugs via increased or decreased absorption, distribution, metabolism, or excretion (ADME)^13–17^. Knowledge of mRNA expression changes for pharmacogenes has precedence for translating to clinical actionability and adoption into clinical practice, improving successful drug response rates^13–17^. The clinical importance of pharmacogene expression is also evidenced by 483 FDA drug labels warning of drug-drug interactions and genetic variants that alter the activity of pharmacogenes mediating patient exposure to that drug^18^. Diseases can also alter pharmacogene expression, but have been less studied compared to drug-drug interactions and pharmacogenomics. NAFLD is a disease characterized by the deposition of lipids in liver tissue that is accompanied by significant inflammatory signaling. Inflammation alters pharmacogene expression^19–23^ and therefore it is mechanistically plausible that patients with NAFLD would not respond normally to many drugs due to altered pharmacogene-mediated drug disposition.

Existing reports of pharmacogene expression changes in livers of patients with NAFLD is limited to a few studies^24^. Two studies used CYP-targeted real-time PCR^25^ or a whole transcriptome array focusing on absorption, distribution, metabolism, or excretion genes^26^, and their findings indicate that CYP enzyme and transporter mRNA expression is altered in steatosis and NASH. Another study found differences in the abundance of certain pharmacogene proteins in NAFLD-cirrhosis, however the sample size was limited (*n* = 9 cases) and statistically significant conclusions were not made^27^. Other targeted studies found CYP3A4 protein and activity was downregulated and CYP2E1 protein and activity was upregulated in NASH vs. controls^27–29^. Collectively, the findings indicate that certain pharmacogenes may be altered in NASH vs. controls, but pharmacogene expression characterization in other clinically used histological severity markers, like fibrosis and NAFLD activity score (NAS), are still lacking in the literature. Two separate studies reported one pharmacogene, *CYP2C19*, is downregulated in liver fibrosis^30,31^ and NAS^31^, however, pharmacogenes were not the focus and the studies did not detail progressive changes in expression across the spectrum of disease. Currently, there are no studies detailing the range of pharmacogene mRNA expression changes that occur over the spectrum of histological disease measures (NAFLD activity score, NASH grade, and fibrosis stage). Therefore, in this study we comprehensively analyzed the changes in pharmacogene expression across the histological severity spectrum in NAFLD. Our study consists of a larger cohort of liver biopsies, utilizes RNA-seq technology, and utilizes linear regression for 3 clinically defined histological measures of NAFLD severity (NAFLD activity score, NASH grade, and fibrosis stage). Addressing this gap in knowledge regarding progressive pharmacogene expression changes in NAS, fibrosis, and NASH has important implications for improving patient care and for NASH drug development.

Focusing on pharmacogenes is advantageous not only by the potential for clinical actionability, but it also increases the likelihood of identifying true positive associations by avoiding transcriptome-wide multiple-testing corrections. Other gene expression studies have been conducted in NAFLD liver samples^25,26,30–48^, but since their focus was transcriptome-wide, they needed to multiply their *p*-values by the number of genes tested (often over 20,000) to obtain Bonferroni-corrected significance values. Because of this, many pharmacogene associations remain undiscovered and buried in raw data. Therefore, a second aim of our study was to validate our findings by conducting a meta-analysis in data obtained from 16 studies of transcriptome-wide hepatic gene expression including our own. This 2-step methodologic approach provides a higher level of statistical rigor to demonstrate the magnitude of the validated pharmacogene expression changes in NAFLD. Thus, in this study we determine if NAFLD patients could be at risk of altered drug response due to changes in hepatic expression of genes that mediate drug disposition across histological NAFLD severity.

## Results

We conducted linear regression analyses to identify associations between liver expression of 255 pharmacogenes and NAS, fibrosis stage, and presence of steatohepatitis in 93 individual liver biopsies. The majority of the pharmacogene mRNA expression values that correlated with the histological phenotypes were unskewed indicating normal distribution. All phenotypes showed good representation across the range of disease severity. Table 1 shows the distribution of these disease phenotypes along with other important clinical and demographic information. In total, we identified 37 pharmacogene-NAFLD severity associations that were statistically significant (*p* ≤ 0.05) after Bonferroni correction (*p*-value multiplied by 255). We also performed t-tests for disease groups (above or below the disease threshold as described in the methods section) to identify 6 additional genes that were statistically significant after Bonferroni correction. Of the significant differentially expressed genes among the t-tests, 82% were also significant in the linear regressions. We also identified 17 genes that were commonly significant for all 3 disease subgroups after Benjamini-Hochberg multiple-testing correction. *CYP2C19* had the strongest effect size of any pharmacogene.

**Table 1:** Demographic and clinical information

	Total	NA^	NASH Diagnosis (steatohepatitis grade)				NAS (NAFLD activity score)										Fibrosis Stage					
			0	1	2	NG*	0	1	2	3	4	5	6	7	8	NG*	0	1	2	3	4	NG*
Total	93	0	23	12	54	4	5	6	15	14	18	18	12	5	0	0	27	26	15	13	6	6
Sex																						
Female	55	0	11	8	33	3	4	2	9	4	12	13	8	3	0	0	13	12	9	9	6	6
Male	38	0	12	4	21	1	1	4	6	10	6	5	4	2	0	0	14	14	6	4	0	0
Race																						
Asian	2	0	2	0	0	0	0	0	1	1	0	0	0	0	0	0	2	0	0	0	0	0
Black	4	0	0	0	2	2	0	0	1	0	3	0	0	0	0	0	0	2	0	0	0	2
Hispanic	1	0	0	0	1	0	0	0	0	0	0	0	1	0	0	0	0	1	0	0	0	0
Other	2	0	1	1	0	0	0	1	0	0	1	0	0	0	0	0	2	0	0	0	0	0
White	84	0	20	11	51	2	5	5	13	13	14	18	11	5	0	0	23	23	15	13	6	4
Concomitant Medications, yes (no)																						
statins	20 (57)	16	4 (13)	0 (11)	15 (30)	1 (3)	1 (2)	2 (4)	2 (10)	2 (10)	5 (10)	4 (11)	4 (5)	0 (5)	0 (0)	0 (0)	3 (17)	5 (18)	3 (9)	4 (6)	2 (4)	3 (3)
fishoil	9 (69)	15	1 (16)	1 (10)	7 (39)	0 (4)	0 (3)	1 (5)	0 (12)	1 (11)	2 (13)	3 (13)	2 (7)	0 (5)	0 (0)	0 (0)	2 (18)	3 (20)	1 (11)	2 (9)	0 (6)	1 (5)
metformin	19 (58)	16	2 (15)	0 (11)	15 (30)	2 (2)	1 (2)	2 (4)	1 (11)	2 (10)	4 (11)	4 (12)	2 (6)	3 (2)	0 (0)	0 (0)	2 (18)	8 (15)	3 (9)	3 (8)	1 (4)	2 (4)
thiazolidinediones	4 (73)	16	1 (15)	0 (11)	3 (43)	0 (4)	0 (3)	0 (5)	1 (11)	2 (10)	1 (14)	0 (17)	0 (8)	0 (5)	0 (0)	0 (0)	0 (19)	1 (22)	2 (10)	0 (12)	1 (4)	0 (6)
vitamin E	12 (64)	17	2 (16)	2 (9)	8 (35)	0 (4)	0 (3)	1 (5)	2 (11)	3 (8)	3 (12)	1 (14)	1 (7)	1 (4)	0 (0)	0 (0)	3 (18)	6 (16)	0 (11)	3 (7)	0 (6)	0 (6)
Comorbidities,*^ yes (no)																						
obesity**	69 (22)	2	12 (10)	5 (7)	48 (5)	4 (0)	2 (3)	1 (5)	11 (4)	8 (5)	15 (3)	17 (1)	10 (1)	5 (0)	0 (0)	0 (0)	12 (14)	21 (4)	13 (2)	11 (2)	6 (0)	6 (0)
diabetes	36 (42)	15	6 (12)	1 (10)	27 (18)	2 (2)	1 (2)	2 (4)	6 (7)	2 (10)	6 (9)	10 (6)	5 (3)	4 (1)	0 (0)	0 (0)	4 (17)	11 (11)	5 (7)	7 (4)	6 (0)	3 (3)
CAD	7 (69)	17	3 (14)	1 (9)	2 (43)	1 (3)	1 (2)	1 (4)	3 (10)	1 (10)	0 (14)	0 (16)	0 (9)	1 (4)	0 (0)	0 (0)	2 (17)	3 (20)	0 (12)	0 (10)	1 (5)	1 (5)
hyperlipidemia	40 (35)	18	9 (7)	3 (7)	28 (18)	0 (3)	0 (2)	2 (2)	7 (6)	8 (4)	7 (8)	9 (7)	6 (2)	1 (4)	0 (0)	0 (0)	10 (8)	12 (10)	5 (8)	6 (5)	4 (2)	3 (2)
hypothyroid	13 (63)	17	2 (15)	2 (9)	8 (37)	1 (2)	0 (2)	0 (5)	2 (11)	3 (9)	3 (12)	3 (13)	1 (8)	1 (3)	0 (0)	0 (0)	4 (16)	1 (21)	3 (9)	2 (9)	1 (5)	2 (3)
HTN	36 (36)	21	6 (11)	1 (9)	27 (15)	2 (1)	0 (2)	0 (5)	7 (6)	5 (5)	6 (8)	10 (5)	5 (3)	3 (2)	0 (0)	0 (0)	4 (15)	16 (6)	5 (5)	5 (5)	3 (3)	3 (2)
Continuous Variables, Mean (SD)																						
Weight, Kg	100 (24)	52	97 (28)	75 (13)	104 (24)	93 (9)	87 (NA)	86 (NA)	86 (15)	93 (6)	97 (28)	109 (27)	94 (10)	130 (41)	NA	NA	96 (29)	95 (12)	130 (34)	107 (29)	83 (20)	89 (6)
Age	50 (13)	0	49 (14)	50 (15)	50 (12)	50 (15)	61 (3)	49 (14)	51 (12)	48 (12)	46 (13)	50 (13)	53 (13)	48 (9)	NA	NA	48 (12)	49 (15)	51 (12)	49 (10)	59 (8)	49 (13)
BMI	32 (9)	4	32 (5)	26 (10)	34 (10)	34 (3)	32 (5)	27 (4)	32 (5)	28 (9)	31 (9)	36 (11)	31 (10)	41 (8)	NA	NA	30 (8)	32 (8)	33 (12)	36 (8)	29 (15)	35 (3)
alt	70 (45)	5	53 (27)	51 (36)	81 (50)	67 (35)	58 (37)	55 (39)	46 (21)	63 (59)	60 (46)	84 (32)	101 (55)	97 (41)	NA	NA	55 (32)	84 (58)	89 (50)	53 (21)	68 (53)	66 (27)
platelets	237 (82)	13	224 (79)	246 (51)	245 (90)	194 (17)	184 (61)	204 (69)	247 (87)	218 (66)	232 (68)	240 (57)	282 (134)	238 (76)	NA	NA	222 (77)	229 (64)	272 (120)	232 (42)	289 (145)	205 (33)

*NG = Not graded, not staged, not scored.

^NA = not available, missing data, unknown.

^**^BMI > 30, or per medical history.

*^The presence of these comorbidities is based on a combination of medical history and medication use in their medical records. For example, if someone is on sulfonylureas we assigned them as having diabetes. Similarly, if someone is taking a statin, then hyperlipidemia is identified, etc.

### NAFLD activity score

**Fig. 1:**
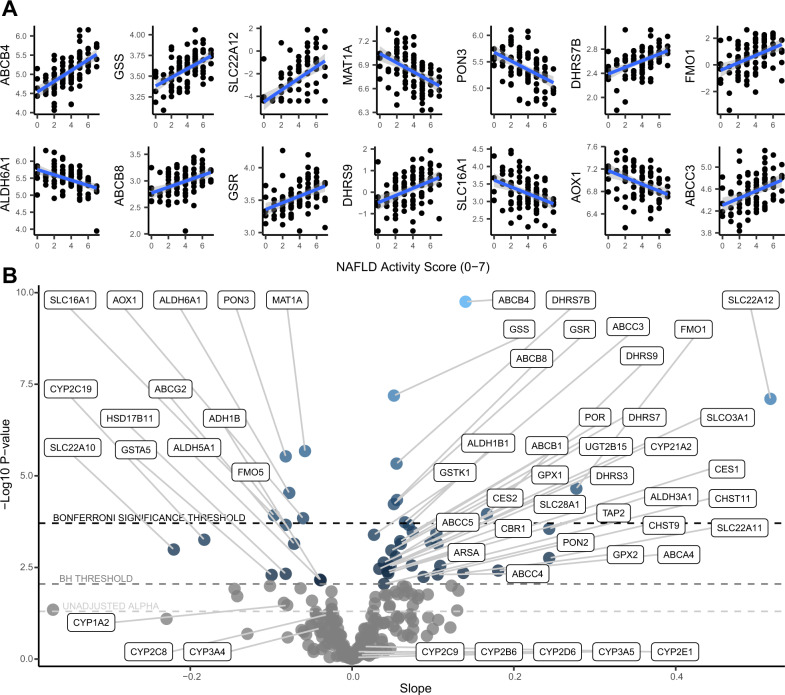
Regression results for NAFLD activity score (*n* = 93). **A** Regression plots of the Bonferroni-adjusted significant correlations between NAS and pharmacogene mRNA expression. Linear trendlines were fit (blue line) with 95% confidence interval region (grey). **B** Volcano plot showing the slopes and p-values for the regressions between NAS and pharmacogene mRNA expression. Horizontal dotted lines correspond to the Bonferroni significance threshold, Benjamini Hochberg (BH) significance threshold, and uncorrected alpha of 0.05, from top to bottom, respectively. Source data are provided in the supplemental materials.

**Fig. 2:**
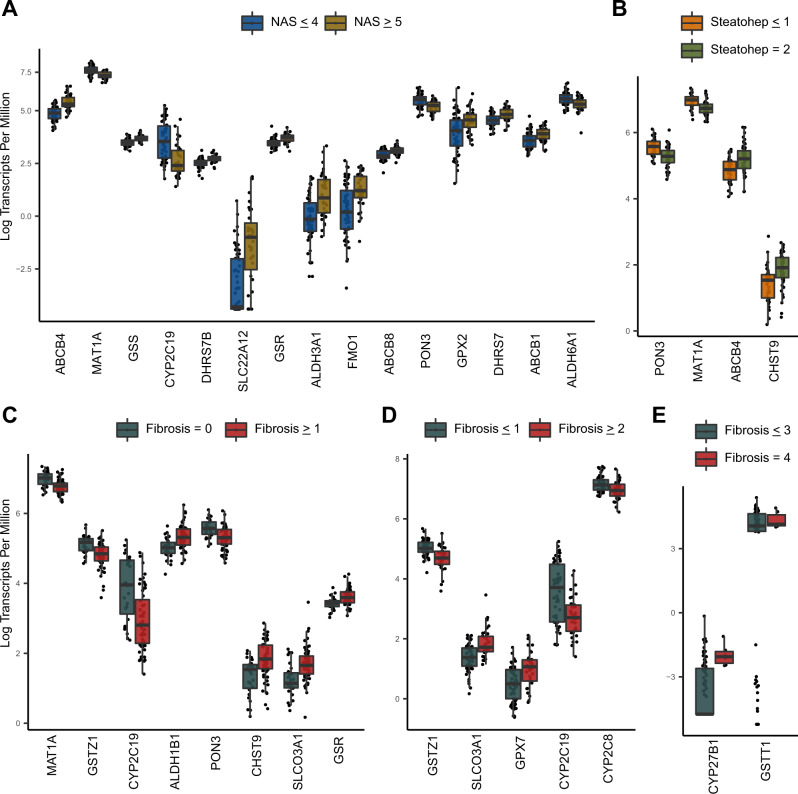
Boxplots showing Bonferroni-adjusted significant t-test results. Differences are based on two-sided Welches t-tests. The x-axis is ordered from left to right by *p*-value. All *p*-values in this figure are below 0.0002. Standard boxplot features (lower quartile, median, upper quartile) are used as defined by ggplot2 version 3.3.6. **A** Expression differences in pharmacogenes between NAS groups (*n* = 93). **B** Expression differences in pharmacogenes between steatohepatitis groups (*n* = 89). **C–E** Expression differences in pharmacogenes between fibrosis groups (*n* = 87). Source data are provided in the supplemental materials.

The 14 Bonferroni-adjusted statistically significant individual pharmacogene regressions for NAFLD activity score (NAS) are shown in Fig. 1A. The NAS within our cohort ranged from 0–7, within a possible histological range of least severe (0) to most severe (8) disease. The direction of effect was positive (upward) for 9 of the pharmacogenes and negative (downward) for the other 5. The result with the lowest *p*-value was a positive correlation for *ABCB4* with an R^2^ of 36% (correlation coefficient 0.6). *ABCB8*, *ABCC3*, and *SLC22A12* were also significantly upregulated, while *AOX1* and *SLC16A1* were significantly downregulated with increasing NAS. Table 2 shows the full list and the individual estimates from each regression. To better visualize and compare these results, we plotted the slopes and *p*-values for all 255 associations in the volcano plot shown in Fig. 1B. The downregulation of *CYP2C19* and *ABCG2* met the less stringent multiple-comparisons correction (Benjamini-Hochberg). Although not statistically significant, *CYP3A4*, *CYP1A2*, and *CYP2C8* trended downwards. Other major CYP enzymes do not appear to be robustly affected by NAFLD severity. Upregulated genes that met the less stringent threshold are *CES1*, *CES2*, several ABC transporters (*ABCB1*, *ABCC5*, *ABCC4*, *ABCA4*), SLC transporters (*SLC03A1*, *SLC28A1*, *SLC22A11*), *CYP21A2*, and *UGT2B15*. In addition to the regression analysis, we identified significant differences in pharmacogene mRNA expression levels between the most severe disease (NAS 5–8) vs. the less severe disease (NAS 0–4), shown in Fig. 2A. This dichotomous analysis identified the Bonferroni-adjusted significant downregulation of *CYP2C19*.

**Table 2:** Bonferroni-adjusted significant linear regression correlation estimates between disease sub-groups and pharmacogenes

Gene	Slope	Unadjusted *p* value	R-squared	Adjusted *p* value (Bonferroni)	Disease sub-group
ABCB4	0.14	1.77E-10	0.36	4.51E-08	NAS
GSS	0.05	6.46E-08	0.28	1.65E-05	NAS
SLC22A12	0.52	7.92E-08	0.27	2.02E-05	NAS
MAT1A	−0.06	2.11E-06	0.22	5.37E-04	NAS
PON3	−0.08	2.94E-06	0.21	7.50E-04	NAS
DHRS7B	0.05	4.64E-06	0.21	1.18E-03	NAS
FMO1	0.28	2.22E-05	0.18	5.67E-03	NAS
ALDH6A1	−0.08	2.92E-05	0.18	7.45E-03	NAS
ABCB8	0.06	4.55E-05	0.17	1.16E-02	NAS
GSR	0.05	5.89E-05	0.16	1.50E-02	NAS
DHRS9	0.17	1.10E-04	0.15	2.80E-02	NAS
SLC16A1	−0.10	1.19E-04	0.15	3.04E-02	NAS
AOX1	−0.06	1.43E-04	0.15	3.66E-02	NAS
ABCC3	0.07	1.85E-04	0.14	4.72E-02	NAS
GSTZ1	−0.17	5.51E-09	0.33	1.40E-06	Fibrosis
SLCO3A1	0.24	7.34E-09	0.33	1.87E-06	Fibrosis
ABCC4	0.27	3.33E-07	0.27	8.50E-05	Fibrosis
MAT1A	−0.09	3.76E-07	0.26	9.60E-05	Fibrosis
GSTP1	0.20	7.95E-07	0.25	2.03E-04	Fibrosis
CYP2C19	−0.37	1.90E-06	0.24	4.85E-04	Fibrosis
CHST9	0.22	2.23E-06	0.23	5.68E-04	Fibrosis
GPX7	0.24	2.96E-06	0.23	7.54E-04	Fibrosis
SLC28A3	0.35	3.28E-06	0.23	8.35E-04	Fibrosis
SOD3	0.36	5.62E-06	0.22	1.43E-03	Fibrosis
CFTR	0.40	9.04E-06	0.21	2.31E-03	Fibrosis
PDE3A	0.24	1.56E-05	0.20	3.98E-03	Fibrosis
SLC2A4	−0.24	1.67E-05	0.20	4.26E-03	Fibrosis
DHRS7B	0.08	2.26E-05	0.19	5.75E-03	Fibrosis
ABCC1	0.22	2.71E-05	0.19	6.92E-03	Fibrosis
ALDH3B1	0.14	2.86E-05	0.19	7.28E-03	Fibrosis
CYP1B1	0.15	4.35E-05	0.18	1.11E-02	Fibrosis
SLC22A17	0.19	6.17E-05	0.17	1.57E-02	Fibrosis
CHST4	0.33	6.28E-05	0.17	1.60E-02	Fibrosis
HSD17B14	−0.26	6.45E-05	0.17	1.65E-02	Fibrosis
SLC6A6	0.23	7.07E-05	0.17	1.80E-02	Fibrosis
CHST10	0.27	1.29E-04	0.16	3.30E-02	Fibrosis
CHST3	0.20	1.35E-04	0.16	3.45E-02	Fibrosis
ALDH1A3	0.23	1.40E-04	0.16	3.58E-02	Fibrosis
MAT1A	−0.14	2.28E-07	0.27	5.81E-05	Steatohepatitis
CHST9	0.31	4.18E-06	0.22	1.07E-03	Steatohepatitis
CYP2C19	−0.50	5.98E-06	0.21	1.53E-03	Steatohepatitis
PON3	−0.16	3.44E-05	0.18	8.77E-03	Steatohepatitis
GSS	0.09	5.04E-05	0.17	1.29E-02	Steatohepatitis
ABCB4	0.21	5.78E-05	0.17	1.47E-02	Steatohepatitis
SLCO3A1	0.24	1.34E-04	0.16	3.42E-02	Steatohepatitis
AOX1	−0.13	1.70E-04	0.15	4.35E-02	Steatohepatitis

*NAS* Non-Alcoholic Fatty Liver Disease Activity Score.

### Fibrosis stage

**Fig. 3:**
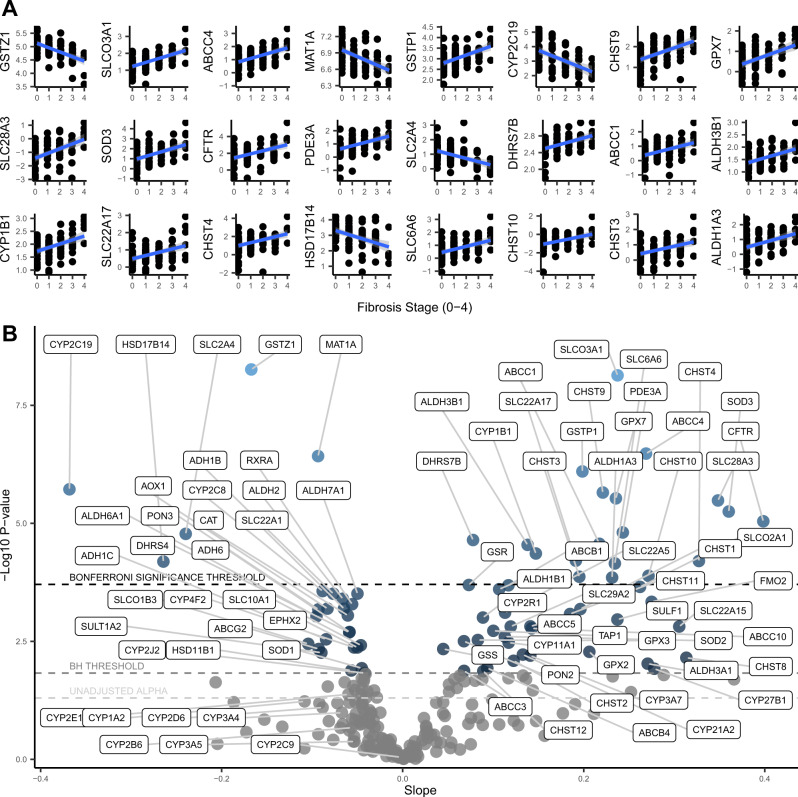
Regression results for fibrosis stage (*n* = 87). **A** Regression plots of the Bonferroni-adjusted significant correlations between fibrosis stage and pharmacogene mRNA expression. Linear trendlines were fit (blue line) with 95% confidence interval region (grey). **B** Volcano plot showing the slopes and *p*-values for the regressions between fibrosis stage and pharmacogene mRNA expression. Horizontal dotted lines correspond to the Bonferroni significance threshold, Benjamini Hochberg (BH) significance threshold, and uncorrected alpha of 0.05, from top to bottom, respectively. Source data are provided in the supplemental materials.

We also conducted linear regression analyses to evaluate pharmacogene mRNA expression levels across liver fibrosis stages (ranging from 0–4). There were 24 Bonferroni-adjusted statistically significant associations, with 19 positive, and 5 negative (Fig. 3A). The result with the lowest *p*-value was a negative correlation for *GSTZ1* with an R^2^ of 33% (correlation coefficient −0.57). *CYP2C19* also had a strong negative correlation with an R^2^ of 24% (correlation coefficient −0.49), and *SLC2A4* was also significantly downregulated. *CYP1B1*, *ABCC1*, *ABCC4*, *SLCO3A1*, *SLC6A6*, and *SLC22A17* were significantly upregulated. Table 2 shows the full list and the individual estimates from each regression. To better visualize and compare these results, we plotted the slopes and *p*-values for all 255 associations in the volcano plot shown in Fig. 3B. The downregulation of *CYP2C8*, *SLCO1B3*, *SLC22A1*, *SLC10A1*, *CYP4F2*, *CYP2J2*, *AOX1*, and *ABCG2* met the less stringent multiple-comparisons correction (Benjamini-Hochberg). *CYP1A2* and *CYP3A4* trended downward (consistent with the NAS analysis) but were not statistically significant. The upregulation of several minor CYP enzymes (*CYP3A7*, *CYP11A1*, *CYP21A2*, *CYP27B1*, *CYP2R1*), ABC transporters (*ABCB1*, *ABCC10*, *ABCC5*, *ABCB4*, and *ABCC3*), and SLC transporters (*SLC22A5*, *SLCO2A1*, *SLC29A2*, *SLC22A15*) met the less stringent multiple corrections threshold. We also conducted t-tests using fibrosis thresholds of 0 vs. ≥ 1 (Fig. 2C), ≤ 1 vs. ≥ 2 (Fig. 2D), and ≤ 3 vs. 4 (Fig. 2E) and identified an additional 6 Bonferroni-adjusted significant associations, including *CYP2C8*.

### NASH grade

**Fig. 4:**
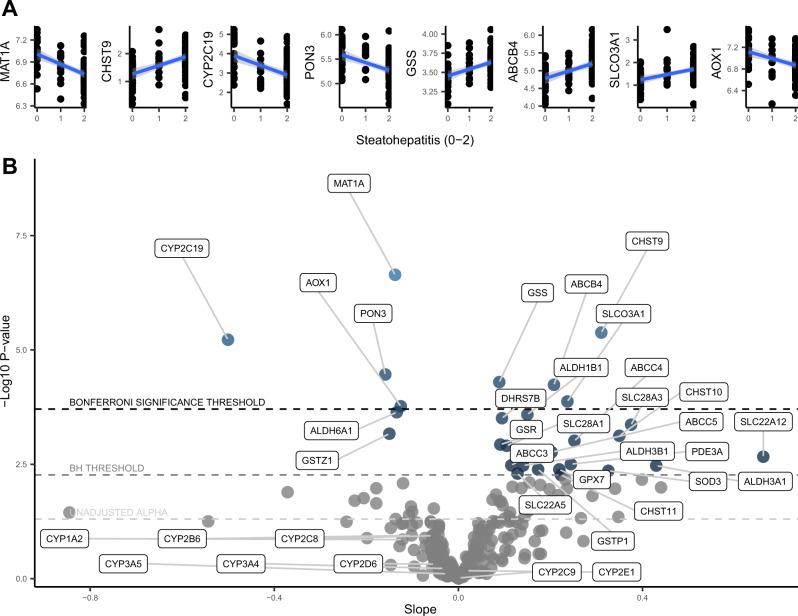
Regression results for steatohepatitis grade (*n* = 89). **A** Regression plots of the Bonferroni-adjusted significant correlations between fibrosis grade and pharmacogene mRNA expression. Linear trendlines were fit (blue line) with 95% confidence interval region (grey). **B** Volcano plot showing the slopes and *p* values for the regressions between steatohepatitis grade and pharmacogene mRNA expression. Horizontal dotted lines correspond to the Bonferroni significance threshold, Benjamini Hochberg (BH) significance threshold, and uncorrected alpha of 0.05, from top to bottom, respectively. Source data are provided in the supplemental materials.

The final disease phenotype we assessed was steatohepatitis (NASH), categorized as 0, 1, or 2, representing no steatohepatitis, borderline steatohepatitis, or definite steatohepatitis, respectively. There were 8 Bonferroni-adjusted statistically significant pharmacogene-steatohepatitis associations, with 4 positive, and 4 negative (Fig. 4A). The result with the lowest *p*-value was observed for *MAT1A* with a negative correlation and an R^2^ of 27% (correlation coefficient −0.52). *CYP2C19* and *AOX1* were also significantly downregulated. *ABCB4* and *SLCO3A1* were significantly upregulated. Table 2 shows the full list and the individual estimates from each regression. To better visualize and compare these results, we plotted the slopes and *p*-values for all 255 associations in the volcano plot shown in Fig. 4B. The upregulation of ABC transporters (*ABCC4*, *ABCC3*, *ABCC5*) and SLC transporters (*SLC22A5*, *SLC28A1*, *SLC28A3*, *SLC22A12*) met the less stringent multiple-comparisons correction (Benjamini-Hochberg). *CYP1A2*, *CYP3A4*, and *CYP2C8* trended downward (consistent with the NAS and fibrosis analysis) but were not statistically significant. We also conducted t-tests using steatohepatitis thresholds of ≤ 1 vs. 2 (Fig. 2B), finding no additional Bonferroni-adjusted significant associations beyond what was found in the regressions. The full analysis results for each pharmacogene for each NAFLD measure are provided in the Supplementary Data 1–11.

### Pharmacogene changes shared between NAS, fibrosis, and NASH

**Fig. 5:**
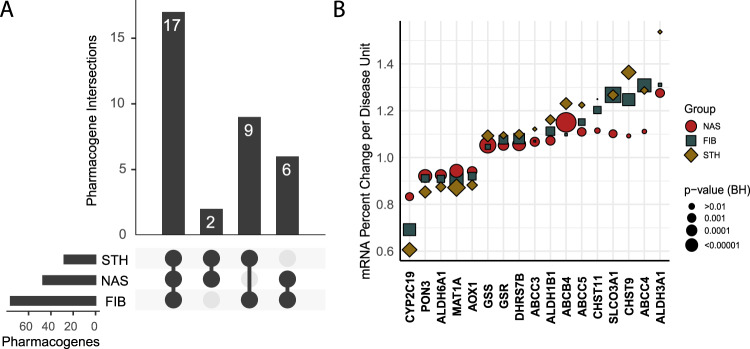
Comparison of the Benjamini-Hochberg significant pharmacogene expression changes between disease subgroups. **A** Upset plot showing the commonality in significantly changed pharmacogenes between disease subgroups. **B** Scatter plot showing mRNA percent change per unit of disease for the 17 pharmacogenes in common between all disease subgroups. For example, the plot shows that with each 1 unit increase in fibrosis stage, *CYP2C19* transcript abundance decreases to 69% of the expression level at the prior stage. For each 1 unit increase in NAFLD activity score, *CYP2C19* transcript abundance decreases to 83% of the expression level at the prior score. For each 1 unit increase in steatohepatitis grade, *CYP2C19* transcript abundance decreases to 61% of the expression level at the prior grade. FIB fibrosis, NAS NAFLD activity score, STH steatohepatitis, BH Benjamini-Hochberg. Source data are provided in the supplemental materials.

Because of the correlation between the 3 phenotypes of interest (NAS, fibrosis, and NASH) (Supplementary Figure 1, and Supplementary data 15, 16, and 17), it is useful to identify the pharmacogenes that are commonly altered by all 3 of these measures. Figure 5A shows an upset plot^49,50^ (similar to a Venn diagram) of the Benjamini-Hochberg significant pharmacogene associations, demonstrating that 17 pharmacogenes are changed in all 3 disease subgroups. Figure 5B shows the percent fold change values for the 17 common pharmacogenes (numerical data provided in Table 3). *CYP2C19* showed the largest downregulation that was significant among all three disease measures. This analysis shows that with every 1 unit increase in fibrosis stage, *CYP2C19* transcript abundance decreases to 69% that of the prior stage; reducing *CYP2C19* mRNA expression levels by 77% in individuals with stage 4 fibrosis compared to 0. With every 1 unit increase in NAS, *CYP2C19* transcript abundance decreases to 83% that of the prior score; reducing *CYP2C19* mRNA expression levels by 73% in individuals with an NAS of 7 compared to 0. As an individual progresses from no-NASH to borderline NASH to definite NASH, *CYP2C19* transcript abundance decreases to 61% that of the prior grade; reducing *CYP2C19* mRNA expression levels by 63% in individuals with NASH compared to those without NASH.

**Table 3:** Regression estimates for the 17 pharmacogenes commonly changed in all 3 disease subgroups, converted to percent fold change per disease unit

Gene	Fibrosis FC per disease unit	Fibrosis BH *p* value	NAS FC per disease unit	NAS BH *p* value	Steatohepatitis FC per disease unit	Steatohepatitis BH *p* value
CYP2C19	0.69	8.08E-05	0.83	6.73E-03	0.61	5.08E-04
ALDH6A1	0.91	5.64E-03	0.93	9.31E-04	0.87	6.53E-03
MAT1A	0.91	2.40E-05	0.94	1.34E-04	0.87	5.81E-05
PON3	0.91	4.47E-03	0.92	1.50E-04	0.85	2.19E-03
AOX1	0.92	4.47E-03	0.94	2.81E-03	0.88	5.43E-03
GSS	1.05	1.89E-02	1.05	6.74E-06	1.09	2.46E-03
ABCC3	1.07	4.60E-02	1.07	3.37E-03	1.12	3.74E-02
GSR	1.08	2.01E-03	1.05	1.50E-03	1.10	1.86E-02
DHRS7B	1.08	4.11E-04	1.06	1.97E-04	1.10	7.20E-03
ABCB4	1.10	4.21E-02	1.15	4.51E-08	1.23	2.46E-03
ALDH1B1	1.11	2.27E-03	1.07	3.46E-03	1.16	6.58E-03
ABCC5	1.15	8.33E-03	1.11	4.91E-03	1.22	2.44E-02
CHST11	1.20	5.30E-03	1.12	2.13E-02	1.25	4.92E-02
CHST9	1.25	8.11E-05	1.09	3.31E-02	1.36	5.08E-04
SLCO3A1	1.27	9.35E-07	1.10	7.01E-03	1.27	4.88E-03
ABCC4	1.31	2.40E-05	1.11	2.99E-02	1.29	1.65E-02
ALDH3A1	1.31	3.47E-02	1.28	4.18E-03	1.54	3.74E-02

*FC* fold change, *NAS* NAFLD activity score, *BH* Benjamini Hochberg.

### Covariate analysis

To determine if the *CYP2C19* downregulation could be better explained by something other than NAFLD, we conducted single linear regressions of *CYP2C19* mRNA abundance with each possible independent variable in our clinical and demographic data (Supplementary Data 12). We found that only the histological markers of NAFLD (fibrosis stage, steatohepatitis, hepatocyte ballooning, and NAS) were statistically significant after Bonferroni multiple corrections (Supplementary Data 13). However, several other factors like AST, ALT, age, metformin use, and diabetes, were possibly associated (uncorrected *p*-value <0.05). To determine if these factors, and sex, could have influenced our results, we performed linear regressions between *CYP2C19* mRNA abundance and each NAFLD phenotype with and without correcting for each of these respective covariates. The change in *CYP2C19* slope before and after covariate correction was measured, and no covariate affected the *CYP2C19* slope by more than 16%, indicating the robustness of the association between the histological NAFLD phenotypes and *CYP2C19* (Supplementary Data 14). This information collectively suggests that metabolic comorbidities like obesity and diabetes, which are highly correlated with NAFLD, are not the primary drivers of the observed *CYP2C19* downregulation. However, it is likely that these comorbidities contribute additional information that, when combined, can improve the characterization of *CYP2C19* downregulation. To provide evidence towards the most important features underlying the *CYP2C19* downregulation, we used a backward elimination approach, narrowing to only remaining coefficients with *p*-values <0.05 in a multiple linear regression model. From this analysis, the following factors explain 39% of the variability in *CYP2C19* mRNA abundance: fibrosis stage, age, metformin use, and ALT (Supplementary Data 18). This model is of limited actionability until it is optimized and validated, but it shows that the additional factors increase the variability explained from 24% (fibrosis only) to 39% (after adding age, metformin use, and ALT).

Importantly, NAS and steatohepatitis were dropped from the multiple regression model, suggesting worsening fibrosis as possibly the most important histological NAFLD feature in describing *CYP2C19* downregulation. We tested this further by re-running the regressions of NAS and steatohepatitis in each fibrosis stage separately (results provided in Supplementary Data 3 and 6), showing that these factors are not associated with *CYP2C19* expression outside of the context of worsening fibrosis. An additional multiple regression model was tested by including just fibrosis stage, NAS, and steatohepatitis together. These results (Supplementary Data 19) show that only fibrosis stage and steatohepatitis diagnosis contribute a meaningful effect size in describing the expression of *CYP2C19*. Further, only fibrosis stage was statistically significant in this analysis.

### NAFLD-CYP2C19 meta analysis

**Fig. 6:**
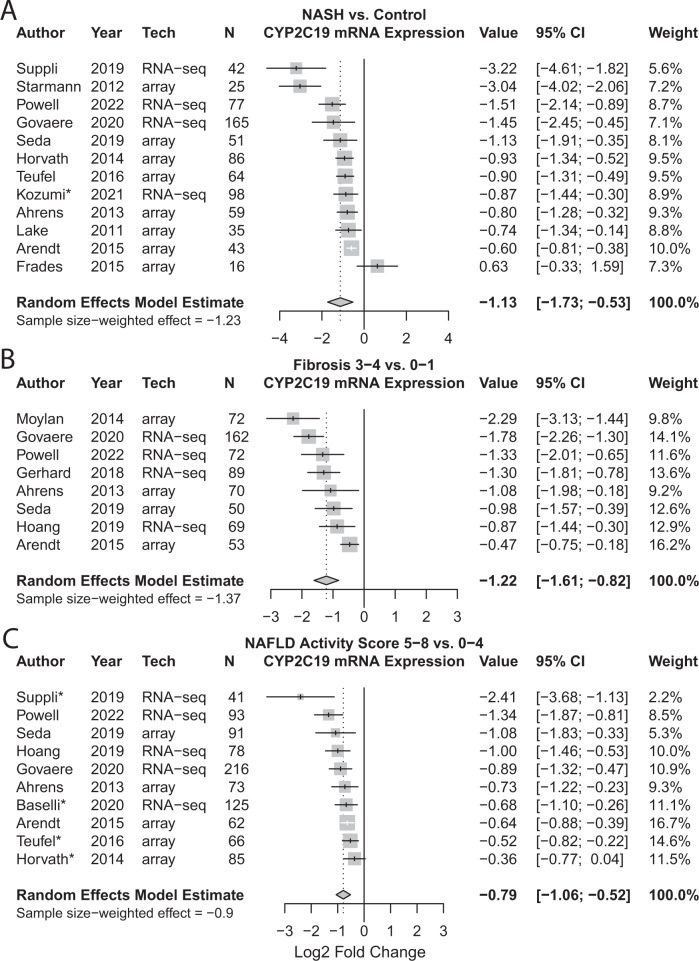
Forest plots demonstrating the meta-analysis effect of NAFLD on *CYP2C19* mRNA expression. Random effects model estimates, weighted based on inverse variance, are provided in bold. Sample size-weighted estimates (effects) are also provided. **A** NASH vs. no-NASH control. Note, studies marked with an asterisk (*) did not have a healthy control group, therefore subjects labelled “NAFL” were used as the comparator. **B** Fibrosis 3-4 vs. 0-1. **C** NAS 5-8 vs. 0-4. Note, for studies marked with an asterisk (*) NAS was not explicitly given therefore the following analyses are included instead: Suppli et al, Teufel et al, and Horvath et al subjects labelled “NAFLD” were compared to healthy controls, Baselli et al subjects labelled “severe NAFLD” were compared to “mild-no NAFLD”. Source data for our study are provided in the supplemental materials and data from other studies is available in GEO.

Because *CYP2C19* was consistently downregulated across three disease severity phenotypes in our study, and due to its actionability in personalized therapy for CYP2C19 substrate drugs like clopidogrel, we conducted a meta-analysis using 16 studies that measured *CYP2C19* expression in NAFLD. Figure 6 demonstrates that *CYP2C19* is consistently downregulated in 15 of 16 studies, validating our findings. The meta-analysis model indicates a log2 fold change of −1.13 in NASH vs. control (Fig. 6A), a value translating to an expression decrease to 46%. Fibrosis stage 3–4 vs. 0–1 indicates a log2 fold change of −1.22 (Fig. 6B), translating to an expression decrease to 43%. NAS 5–8 vs. 0–4 indicates a log2 fold change of −0.79 (Fig. 6C), translating to an expression decrease to 58%. These meta-analysis values validate our findings.

## Discussion

In this study, we screened for altered pharmacogene expression across 93 individual liver biopsies obtained from participants with varying histological stages of NAFLD severity. Our results clearly demonstrate that expression levels of several pharmacogenes are significantly associated with the histological severity of NAFLD. Notably, *CYP2C19* was severely downregulated. By conducting a meta-analysis in 16 similar studies, we validate that *CYP2C19* is strongly downregulated in NASH and advanced fibrosis.

Our study used the current gold standard RNA-seq technology; however, it does have some potential limitations. RNA-seq more readily measures high abundance transcripts, therefore the percent fold changes we report could be affected by this bias. However, since our fold change analyses compare the same gene with similar order-of-magnitude expression levels, this measurement bias is not a major concern. Another potential limitation is the conservative approach taken with the Bonferroni multiple-testing correction. Bonferroni unduly penalizes the statistical significance of results when factors between tests are not completely independent. Since changes in the expression of one gene could have downstream effects on another genes expression, these changes are not all necessarily independent and therefore the false discovery rate (FDR) Benjamini-Hochberg correction can also be appropriate when interpreting results. A limitation of our meta-analysis is that it was not based on a systematic review for which a protocol was published (rather a narrative review). We also did not conduct sensitivity analyses. Despite these limitations, the meta-analysis accomplished our goal of assessing the repeatability and inferring the magnitude of *CYP2C19* change across a variety of other NAFLD cohorts.

Medications such as rifampin, efavirenz, and ritonavir can induce CYP enzyme expression, including CYP2C19^51^. Our analyses would likely be improved by accounting for patients taking these medications, however, this information is not available for our cohort. Despite this limitation, it is unlikely that our findings of CYP2C19 downregulation could be attributed to medication usage unless for some reason healthier patients tended to take more rifampin, efavirenz, or ritonavir. We do not have reason to suspect this in our patient population nor in the other 15 studies in the meta-analysis. Another limitation of our study is that we did not consider the effect of genotype on mRNA abundance. Expression of functional *CYP2C19* is increased by the CYP2C19*17 allele and decreased by CYP2C19*2 and *3 variants^52–54^. The expression changes we found for *CYP2C19* and other pharmacogenes would likely have been more precisely characterized by including known loss-of-function genotypes in the analysis, but since genotypes are unlikely to cluster in severe NAFLD patients, this is not a major limitation. We also recognize there are other ways to classify steatohepatitis. Therefore, we also performed pharmacogene regressions for NASH categorized by the SAF (steatosis, activity, fibrosis) method^55^, and found the slopes were highly correlated with our steatohepatitis results using the CRN method (Pearson correlation coefficient 0.88, Supplementary Data 9).

Another potential limitation of our study is that *CYP2C19* mRNA expression levels may not necessarily correlate with CYP2C19 enzyme abundance. However, Fisher et al. demonstrated that liver *CYP2C19* mRNA downregulation in NASH (*p* = 0.193) corresponded to a significant decrease in liver CYP2C19 enzyme abundance (*p* = 0.01)^25^, indicating *CYP2C19* mRNA is a good surrogate measure for CYP2C19 enzyme abundance. Additionally, the Human Protein Atlas demonstrates corresponding high levels of mRNA and protein expression of CYP2C19 in the liver, further supporting mRNA as a good surrogate measure for enzyme abundance^56^. For certain *CYP2C19* genotypes, reduced mRNA expression is the mechanism behind the altered clinical effect of drugs that are CYP2C19 substrates, providing further evidence^57^. Lastly, NAFLD was recently correlated with 60% lower omeprazole metabolism (a CYP2C19 substrate)^3^, and a 28% decrease in CYP2C19 protein^58^, which is broadly consistent with the expected effect of decreased *CYP2C19* mRNA.

Our results have translational significance, as CYP2C19 is the key enzyme in the bioactivation of the clopidogrel prodrug to its active metabolite, and dysfunction of this activation pathway is known to significantly impair response to clopidogrel^59–61^. Pharmacogenomic studies have shown that when correctly transcribed *CYP2C19* is halved due to heterozygosity of the CYP2C19*2 allele, the bioactivation of clopidogrel is significantly reduced and the antiplatelet efficacy is also significantly decreased^13,17,53^. Because of this, clinical pharmacogenomic guidelines recommend choosing a different antiplatelet drug for CYP2C19*2 carriers^13,17^. Our data demonstrate that NAFLD can have a similar magnitude of effect on *CYP2C19* expression as compared to CYP2C19*2. Even a moderate progression of NAFLD (i.e. NAS from 0 to 4, fibrosis stage from 0 to 2, or steatohepatitis grade 0 to 1) results in a decrease of *CYP2C19* to around 50%. Decreased CYP2C19 abundance is especially relevant to the NAFLD patient population due to their cardiovascular comorbidities and therefore the increased importance of antiplatelet therapy. Based on our findings of decreased *CYP2C19* expression and the clinical actionability of CYP2C19*2, it is logical to suggest that NAFLD patients are at increased risk for clopidogrel treatment failure and therefore more cardiovascular morbidity. Upon subsequent literature review, we identified a study that demonstrated hepatosteatosis was significantly associated with a lack of clopidogrel anti-platelet effect, further supporting our hypothesis that *CYP2C19* downregulation puts NAFLD patients at increased risk for clopidogrel treatment failure^62^. Further translational research into this area will need to keep in mind that patients with NAFLD can have altered platelet homeostasis, and changing to more potent antiplatelet therapy (i.e., the alternatives to clopidogrel) may contribute to a higher risk of bleeding and will need to be weighed against the risk of low clopidogrel efficacy.

It is especially important to consider special populations like NAFLD because large randomized controlled clinical drug trials are not designed to conclude efficacy and safety for subgroups. Among four big trials studying clopidogrel use (CURE, CAPRIE, CHARISMA, and CLARITY-28)^10–12,63^, none conducted NAFLD subgroup analyses and one of these trials even excluded patients with hepatic insufficiency. Therefore, the approved dosages, efficacy, or safety findings from these pivotal studies may not be representative of people with NAFLD. However, these trials did conduct other subgroup analyses. Both CURE and CHARISMA found diabetes to trend in the direction of less clopidogrel efficacy, though not statistically significant. Additionally, CHARISMA found that obesity, hypertension, and hypercholesterolemia showed similar trends toward lower clopidogrel efficacy, though also not statistically significant. In fact, several studies have shown that patients with diabetes have higher on-clopidogrel platelet reactivity and lower clopidogrel active metabolite compared to controls^64–66^. Diabetes and cardiometabolic diseases are highly associated with NAFLD^5–9^ and therefore these data further support the hypothesis that NAFLD is involved in reduced clopidogrel efficacy through the downregulation of *CYP2C19*.

Besides clopidogrel, CYP2C19 plays a major role in the metabolism of several other drugs including those with narrow therapeutic range (e.g. diazepam, phenytoin, voriconazole, carisoprodol, omeprazole, citalopram, pentamidine, thalidomide, and others)^57^. Thus, our data provide an opportunity to consider personalized treatment of all CYP2C19 substrates in NAFLD patients. To make this a reality, however, the clinical biomarker will need to be carefully chosen. Our results show that fibrosis stage is the strongest association with *CYP2C19* mRNA downregulation. It is possible that hepatic necroinflammation, as measured by NAS or steatohepatitis, is associated with *CYP2C19* downregulation because it accompanies worsening fibrosis. In contrast, there may still be independent effects of necroinflammation that could not be detangled analytically due to the collinearity with fibrosis. This will need to be further studied for mechanistic conclusions to be reached. We can infer, however, that the mechanism of *CYP2C19* downregulation is not due to transcriptome-wide decreases in expression because there were more genes that were upregulated with worsening fibrosis.

Our results indicate that exposure or pharmacodynamics of drugs that are substrates for other pharmacogenes may be altered in patients with histologically severe NAFLD. Methionine adenosyltransferase 1 A (*MAT1A*) was a robustly downregulated pharmacogene in our data and across other studies^30,33,34,45^. Overexpression of this gene in bladder cancer tumor xenografts has been shown to confer tolerance to gemcitabine^67^ suggesting that NAFLD patients could have increased liver toxicity when treated with gemcitabine due to a decreased abundance of MAT1A. Aldehyde oxidase 1 (*AOX1*) was another consistently downregulated pharmacogene in our data and across the other studies^26,32–34^. The role of aldehyde oxidase enzyme coded by the *AOX1* gene in human drug metabolism is emerging. Substrates for this phase I metabolic enzyme are many, including clonazepam, nifedipine, and ziprasidone^68,69^. Ziprasidone elimination relies heavily on aldehyde oxidase, therefore it is possible that NAFLD patients would exhibit reduced clearance of ziprasidone due to less AOX1 expression.

Glutathione s-transferase pi 1 (*GSTP1*) was robustly upregulated in our data and others^26,32,34,45^. *GSTP1* codes for a glutathione s-transferase enzyme that catalyzes the conjugation of polar glutathione groups to enhance systemic elimination of chemotherapeutic agents and toxic metabolites. This gene is the subject of much research due to its variety of roles, one of which is promotion of chemotherapy resistance^70,71^. It is likely that the phenotype of GSTP1 overexpression in NAFLD patients is multifaceted, but could involve chemotherapeutic resistance in hepatic tumors, and conversely protection from hepatotoxicity for noncancerous liver tissue.

The multidrug resistance-associated protein genes ABCC3, ABCC4, ABCC5 were robustly upregulated in our data, agreeing with previous findings at both mRNA and protein levels^24,27,72^. This suggests that chemotherapy-resistance in hepatocellular carcinoma is likely to be a much larger barrier to effective treatment for NAFLD patients. Another hypothesis arises from these data, that chemotherapy resistance in HCC driven by NAFLD will exhibit intrinsic mechanisms of chemotherapy resistance. While a comprehensive review of NAFLD-associated pharmacogenes is out of scope, we highlighted several examples in which NAFLD patients could be at risk for drug failure based on altered pharmacogene expression. These data-driven hypotheses are especially strong when there is strong evidence of the pharmacogenes role in that drugs response, and when findings are repeatable across studies, as is shown in our meta-analysis.

While large, dedicated studies of drug metabolism in every patient disease group (like NAFLD) could create more personalized treatment regimens, such studies are often not practically feasible. Our analyses address this problem by providing effect sizes for each of our regression estimates and detail the percent change occurring with each step in disease grade or stage. These estimates will support the development of NAFLD-specific treatment decisions. Our regression estimates will also allow physiologically-based pharmacokinetic (PBPK) models to be developed to optimize dosing of drugs with established exposure-efficacy relationships. Our study not only characterizes pharmacogene changes in NAFLD, but also provides strong validation evidence, by meta-analysis, that *CYP2C19* is downregulated in NAFLD. Our goal in clinical pharmacology is to customize disease treatments based on specific characteristics of the individual patient. To that end, our study provides evidence that NAFLD patients have unique pharmacogene expression profiles across the disease spectrum, and these data will contribute to developing more individualized treatments for NAFLD patients.

## Methods

This research was conducted ethically and in accordance with research protocols approved by the Indiana University Institutional Review Board (protocol numbers: 1506218127, 1011003025R008).

Our 2-step data analysis approach used (1) pharmacogene-NAFLD regressions in our cohort of patients with NAFLD and (2) meta-analysis specifically focusing on our identified gene(s) with the strongest signal and the most potential for developing clinical interventions.

### Patient selection

This study was undertaken on liver samples obtained from 93 patients with well-characterized NAFLD who underwent percutaneous liver biopsies for their clinical care. Liver specimens were submitted to the pathology laboratory for histological assessment via hematoxylin-eosin (HE) and Masson’s trichrome staining and also snap frozen and stored at −80 C as part of a research protocol that was approved by the Indiana University Institutional Review Board. All participants signed an informed consent for the liver biopsy procedure by the radiology proceduralists and a separate informed consent for saving a liver specimen for future research purposes.

### Histological assessment

The histological grades of steatosis, inflammation, ballooning, fibrosis stage, NAFLD activity score (NAS), and presence of steatohepatitis (NASH) were assessed across all biopsy samples in a blinded fashion by a single experienced hepatopathologist using the validated NASH Clinical Research Network scoring system^73^.

### RNA Isolation

BioChain Broad Range Total RNA isolation kits were used to isolate RNA (BioChain Institute Inc. Newark, CA). RNA quantity and quality were measured using Nanodrop and Experion RNA StdSens and HighSense analysis kits (BioRad, Hercules, California). RNA samples with Agilent Bioanalyzer RIN > 6.0 were advanced for RNA library generation.

### RNA-seq

RNA libraries were constructed at Covance Genomics Laboratories with Illumina HT Truseq Stranded kits. 93 unique sample libraries, each from one unique patient, proceeded to RNA sequencing. Samples were pooled in groups of 16 with each pool run on the Illumina HiSeq 2500 platform in 4 lanes. Samples were balanced across pools based on distribution of fibrosis stage, NAS score, date of biopsy, age, BMI, date of RNA isolation, RNA quality indicator (RQI), and RNA yield.

### RNA-seq data processing

Raw data QC was performed, including base composition quality, fragment size, mapping read count, 3’ bias, read count mapping breakdown, adapter/phiX content, heterologous organism contamination, and sex/ethnicity prediction concordance. Greater than 100 million reads were obtained per sample and reads were mapped to a reference human transcriptome. The ratio between the highest and lowest total mapped reads per sample was 4.2, indicating good read depth consistency across samples. Each sample (or library) was assessed in 4 or 5 replicates (assays). Data correlation of replicates from the same sample were checked. One replicate from all 434 assays was excluded due to low correlation. Replicates were aggregated into one measurement value per sample per gene. There were 93 unique patients in the final cohort with both phenotype information and RNA-seq data.

The read counts for RNA-seq were normalized to the total read counts across all genes for each sample, multiplied by 1,000,000 and log_*e*_-adjusted (i.e. transcripts per million, TPM). The TPM method is a widely used normalization procedure that effectively corrects for batch differences in read depth without normalizing away, or dampening, the biological signal^74^. Read counts of zero were assigned the lowest nonzero read count for any gene in each sample to enable inclusion of that data upon log transformation. We used R version 4.0.4 for this data processing.

### Data analyses and statistical tests

The RNA-seq and phenotype data were analyzed using scripts which are provided in the Supplemental documents, and broad descriptions of the analyses are provided here. We used R (version 3.6.0, 4.0.2, and 4.0.4) for all analyses. R code was used to conduct the linear regressions and the t-tests. T-tests were two-sided and based on unequal variances. Bonferroni corrections were applied to *p*-values by multiplying by the number of association tests performed, and Benjamini-Hochberg corrections were conducted using the “p-adjust” function in base R. Pharmacogenes were filtered based on a list of 298 pharmacogenes as described by www.pharmaadme.org, accessed in 2021. This list is based on input from seven major pharmaceutical companies as to which genes perform or regulate drug metabolism or transport. From this list, 255 pharmacogenes were present in the data. A list of these genes and other raw data can be found in the Supplemental documents. Missing phenotype grades/stages were removed from the respective analysis, resulting in samples sizes of 93 for NAS, 87 for fibrosis, and 89 for steatohepatitis.

For linear regressions, the histological grades or stages of disease were used as independent variables on a continuous numerical scale for testing the association with each pharmacogene. Each linear regression test consisted of one pharmacogene mRNA level as the dependent variable and one NAFLD histological measure as the independent variable. Fibrosis was staged between stage 0 (no fibrosis) through stage 4 (cirrhosis). NAFLD activity score was scored between 0 through 8. Steatohepatitis was graded as grade 0 (no NASH), grade 1 (borderline NASH), and grade 2 (definite NASH). Regression slopes are log_*e*_-adjusted differences in expression and therefore represent a log_*e*_ fold-change (ratio) per unit increase in the NAFLD variable. These values were converted to percent fold change by exponentiating Euler’s number (~2.71828) to the log_*e*_ fold change value. For the t-tests, expression was compared between the disease groups as follows: NAFLD activity score was split into low (≤4) and high (≥5); fibrosis was split into stage 0 vs. rest, stage 0-1 vs. rest, and stage 4 vs. rest; and steatohepatitis was split into definite vs. borderline/absent. T-tests were performed to provide an orthogonal description of pharmacogene changes using clinically meaningful thresholds of disease severity.

A multiple linear regression model was built to investigate the most important factors in describing *CYP2C19* expression. This approach utilized a backward elimination approach of the clinical and demographic factors in the Supplementary Data 12. Factors were removed from the model until each of the remaining factors had a *p*-value of less than 0.05. Variability explained was measured by unadjusted R-squared.

### Meta-analysis

We conducted literature searches using Google Scholar, without restricting results to any time frame, with the following search terms: NAFLD gene expression liver. From these results we manually reviewed titles or abstracts from the first 300 results, narrowing to 16 studies that conducted gene expression profiling in liver samples from patients with NAFLD. We additionally reviewed references from these studies and searched the gene expression omnibus (GEO) identifying 11 more relevant studies for a total of 27 possible studies to include. The following terms were used for the GEO search: Non-alcoholic fatty liver disease, fibrosis, NAFLD, NASH, steatohepatitis, fatty liver. The GEO results were filtered to: Homo sapiens, data sets, series, and publication date: 2000 − 2021. Lastly, we conducted further manual searches of pubmed, (using search terms: NAFLD gene expression liver), finding no further studies, ensuring our literature search exhausted most or all available studies that conducted gene expression analysis in NAFLD liver tissue.

We focused the meta-analysis on *CYP2C19* because it was the most consistently downregulated pharmacogene in our 93 NAFLD patients and knowledge that this gene is downregulated has a large potential for clinical actionability. Of the 27 identified studies, *CYP2C19* expression data was available publicly for 11 studies^30,32,34,45–48,75–78^. We obtained access to data for 3 additional studies thanks to gracious contributions by the study authors^26,33,79^. We were able to obtain *CYP2C19* differential expression results directly from the supplement of 2 additional studies^80,81^, resulting in a final meta-analysis pool of 17 studies, including our own, with varying phenotypes. One of the supplemental data sets reported FDR-adjusted *p* values using the Benjamini-Hochberg procedure, and we back-calculated using the number of genes and rank order to estimate the non-adjusted p-value^80^.

Datasets available as “GEO2R” sets were analyzed in the online GEO portal for Log2 fold-change using the default settings with Limma (version 3.26.8)^82^. Datasets that were available as raw data were analyzed similarly to the GEO2R default settings, using Oligo (version 1.62.1) and Limma packages. For Datasets that showed consistent read depth across samples we used the log counts-per-million normalization in Limma, and for Datasets with more inconsistent read depths, we used the Voom normalization as recommended in the Limma and Voom documentation. All *p*-values were converted to 95% confidence intervals using the metagen^83^ R package (version 4.9), followed by forest plot generation.

Phenotype data was not the same for each study, therefore we created groups of analyses that could be compared together. We chose phenotypes that represent the 3 histological measures of NAFLD severity (NAS, NASH, and fibrosis) based on criteria that were available in the most number of studies. These categories are as follows: Fibrosis stages 3-4 vs. stages 0-1, NAS 5-8 vs. 0-4, and NASH vs. absence of NASH. Controls (absence of NASH) were defined as either healthy obese, healthy normal-BMI, and in certain analyses where healthy controls were not available, mild disease was used as the control group. These are further specified in the corresponding figure legends. From the 17 studies, one was not included in our meta-analyses because it only reported steatosis values but not NASH diagnosis, NAS, or fibrosis.

In addition to the strict clinical grouping, the interpretability of the meta-analysis results are enhanced by similar quantification of mRNA in liver samples (using either RNA-seq or an array-based method). Since measurements within each study were compared between disease vs. no disease, the relative numbers (fold changes) eliminate any heterogeneity that could be introduced by theoretical differences between RNA-seq and the array methodology. For each meta-analysis we used a random-effect model, calculating the average Log2 fold-change weighted by the commonly used inverse variance method^83,84^. This approach is rigorous because it considers that NAFLD is not necessarily the only influence on *CYP2C19* expression and is superior to a fixed-effect model which would incorrectly assume that the only influence to between-study heterogeneity comes from population sampling^85^. We confirm that the random-effects model is appropriate because the Cochran’s Q statistic was statistically significantly larger than the degrees of freedom in each analysis, indicating there is between-study heterogeneity (Supplementary Data 21). In addition to results provided by the inverse variance weighting approach, we also provide the sample size-weighted means to show consistency with a more intuitive approach.

### Reporting summary

Further information on research design is available in the Nature Portfolio Reporting Summary linked to this article.

## Supplementary information

Supplementary Information

Description of Additional Supplementary Files

Supplementary Data 1-22

Reporting Summary

## Data Availability

The source data contains logTPM pharmacogene RNA-seq values as described in the methods along with the NAFLD histological disease grades/stages/scores. Full RNA-seq gene count data is available under GEO accession number GSE225740. Gene expression data used in the meta-analysis can be found at the data sources provided in Supplementary Data 20 and 22. Source data are provided with this paper.

## Source data

Source Data

## Code availability

All code used for this manuscript can be found at GitHub (https://github.com/Nickpowe/CYP2C19_NAFLD_code.git).
