# Supplementary material for: Clinically important alterations in pharmacogene expression in histologically severe nonalcoholic fatty liver disease
Source: Nat Commun. 2023 Mar 17;14:1474. doi: 10.1038/s41467-023-37209-1 (PMC10020163; doi:10.1038/s41467-023-37209-1)
Supplement: Supplementary file 1 — Supplementary Information [file 41467_2023_37209_MOESM1_ESM.pdf]

SUPPLEMENTARY FIGURE

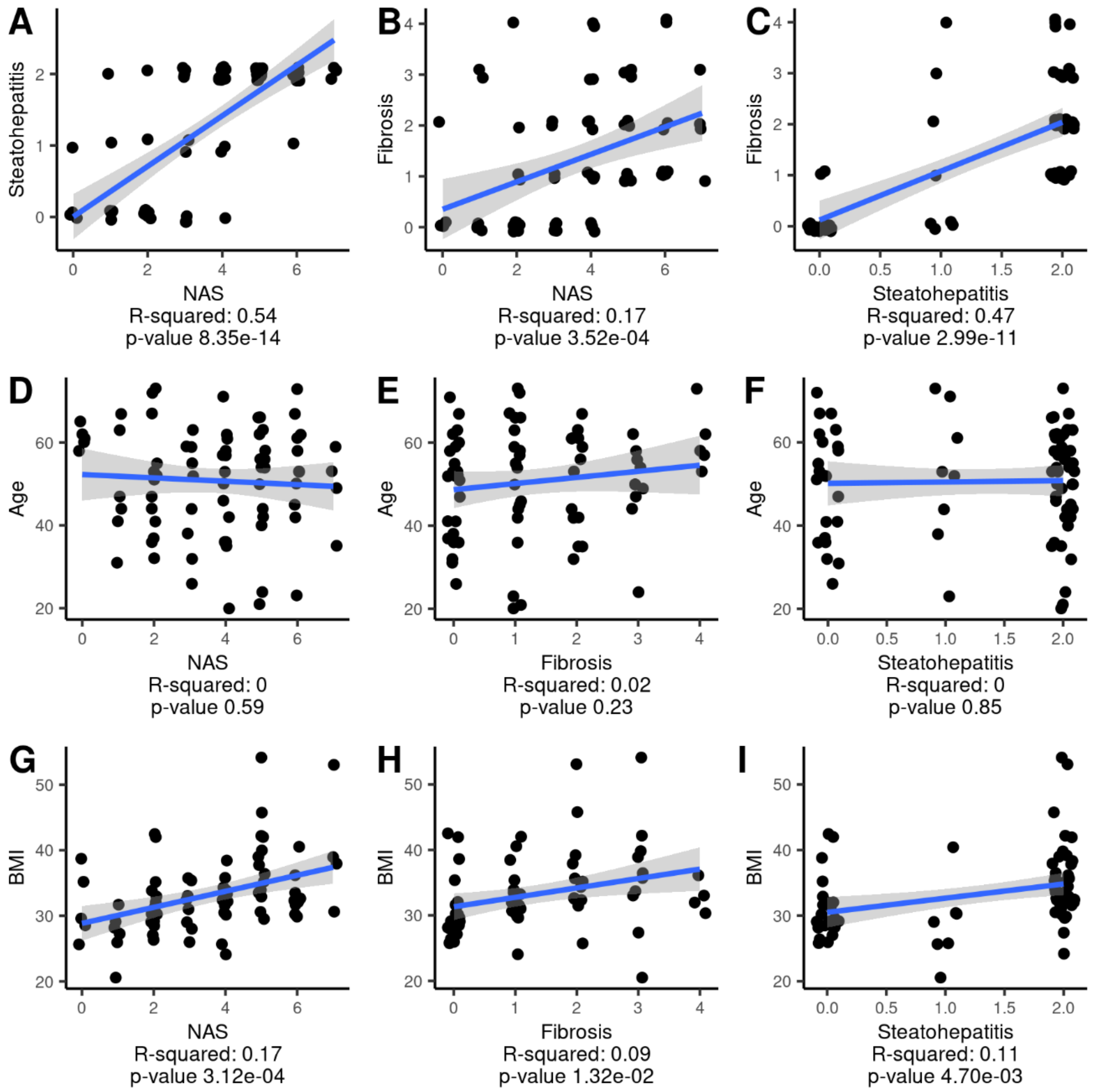

SUPPLEMENTARY Figure 1: Correlation between the 3 types of liver disease (A-C), and regressions between disease phenotypes and age (D-F) and BMI (G-I). Linear trendlines were fit (blue line) with 95% confidence interval region (grey). P-values shown are uncorrected.
