# Supplementary material for: Clinically important alterations in pharmacogene expression in histologically severe nonalcoholic fatty liver disease
Source: Nat Commun. 2023 Mar 17;14:1474. doi: 10.1038/s41467-023-37209-1 (PMC10020163; doi:10.1038/s41467-023-37209-1)
Supplement: Supplementary file 2 — Description of Additional Supplementary Files [file 41467_2023_37209_MOESM2_ESM.pdf]

Description of Additional Supplementary Files

Supplementary Data 1: mRNA~NAS linear regression

Supplementary Data 2: mRNA~NAS linear regression after correcting for Age and Sex

Supplementary Data 3: CYP2C19~NAS regression estimates per fibrosis stage

Supplementary Data 4: mRNA~NASH linear regression

Supplementary Data 5: mRNA~NASH linear regression after correcting for Age and Sex

Supplementary Data 6: CYP2C19~NASH Regressions per fibrosis stage

Supplementary Data 7: mRNA~fibrosis linear regression

Supplementary Data 8: mRNA~fibrosis linear regression after correcting for Age and Sex

Supplementary Data 9: mRNA~SAF diagnosis score linear regression (No Nash, NAFLD, and NASH coded as 0, 1, or 2 respectively)

Supplementary Data 10: All bonferronni significant T-test results

Supplementary Data 11: Benjimini-Hochberg results in common among all 3 NAFLD histological phenotypes

Supplementary Data 12: Demographics

Supplementary Data 13: CYP2C19 associations with covariates

Supplementary Data 14: change in CYP2C19~NAFLD slope with vs. without covariate

Supplementary Data 15: Fibrosis_stage association with covariates

Supplementary Data 16: NAS association with covariates

Supplementary Data 17: Steatohepatitis ("CRN_dx_score") association with covariates

Supplementary Data 18: CYP2C19 Multiple regression model trained on full data, backward elimination to p<0.05

Supplementary Data 19: CYP2C19 Multiple regression model using only Fibrosis, NAS, and steatohepatitis

Supplementary Data 20: Meta-analysis data

Supplementary Data 21: Meta-analysis metrics

Supplementary Data 22: Meta-analysis studies considered
