# Supplementary material for: Clinically important alterations in pharmacogene expression in histologically severe nonalcoholic fatty liver disease
Source: Nat Commun. 2023 Mar 17;14:1474. doi: 10.1038/s41467-023-37209-1 (PMC10020163; doi:10.1038/s41467-023-37209-1)
Supplement: Supplementary file 4 — Reporting Summary [file 41467_2023_37209_MOESM4_ESM.pdf]

nature portfolio

Corresponding author(s): Naga Chalasani

Last updated by author(s): Jan 19, 2023

Reporting Summary

Nature Portfolio wishes to improve the reproducibility of the work that we publish. This form provides structure for consistency and transparency in reporting. For further information on Nature Portfolio policies, see our Editorial Policies and the Editorial Policy Checklist.

Statistics

For all statistical analyses, confirm that the following items are present in the figure legend, table legend, main text, or Methods section.

n/a	Confirmed	
☐	☒	The exact sample size (n) for each experimental group/condition, given as a discrete number and unit of measurement
☐	☒	A statement on whether measurements were taken from distinct samples or whether the same sample was measured repeatedly
☐	☒	The statistical test(s) used AND whether they are one- or two-sided<br>*Only common tests should be described solely by name; describe more complex techniques in the Methods section.*
☐	☒	A description of all covariates tested
☐	☒	A description of any assumptions or corrections, such as tests of normality and adjustment for multiple comparisons
☐	☒	A full description of the statistical parameters including central tendency (e.g. means) or other basic estimates (e.g. regression coefficient) AND variation (e.g. standard deviation) or associated estimates of uncertainty (e.g. confidence intervals)
☐	☒	For null hypothesis testing, the test statistic (e.g. F, t, r) with confidence intervals, effect sizes, degrees of freedom and P value noted<br>*Give P values as exact values whenever suitable.*
☒	☐	For Bayesian analysis, information on the choice of priors and Markov chain Monte Carlo settings
☒	☐	For hierarchical and complex designs, identification of the appropriate level for tests and full reporting of outcomes
☐	☒	Estimates of effect sizes (e.g. Cohen's d, Pearson's r), indicating how they were calculated

Our web collection on statistics for biologists contains articles on many of the points above.

Software and code

Policy information about availability of computer code

Data collection	All code used for this manuscript can be found at github (https://github.com/Nickpowe/CYP2C19_NAFLD_code.git). We permit the re-purposing of this code.<br><br>We used R (version 3.6.0, 4.0.2, and 4.0.4). Datasets available as “GEO2R” sets were analyzed in the online GEO portal for Log2 fold-change using the default settings, (Version info: R 3.2.3, Biobase 2.30.0, GEOquery 2.40.0, limma 3.26.8). Oligo version 1.62.1 and metagen version 4.9 were also used.
Data analysis	We used R (version 3.6.0, 4.0.2, and 4.0.4) for all analyses.

For manuscripts utilizing custom algorithms or software that are central to the research but not yet described in published literature, software must be made available to editors and reviewers. We strongly encourage code deposition in a community repository (e.g. GitHub). See the Nature Portfolio guidelines for submitting code & software for further information.

Data

Policy information about availability of data

All manuscripts must include a data availability statement. This statement should provide the following information, where applicable:

- Accession codes, unique identifiers, or web links for publicly available datasets
- A description of any restrictions on data availability
- For clinical datasets or third party data, please ensure that the statement adheres to our policy

Source data are provided with this paper. The source data contains logTPM pharmacogene RNA-seq values as described in the methods along with the NAFLD histological disease grades/stages/scores. Full RNA-seq gene count data is available under GEO accession number GSE225740 (https://www.ncbi.nlm.nih.gov/geo/query/acc.cgi?acc=GSE225740). Gene expression data used in the meta-analysis can be found at the data sources provided in Supplementary Data 20 and 22.

Human research participants

Policy information about studies involving human research participants and Sex and Gender in Research.

Reporting on sex and gender	Findings do not apply to only one sex. Sex was determined from patient's electronic medical records. Gender was not considered.
Population characteristics	Table 1 provides a detailed breakdown of the clinical and demographic data.
Recruitment	This study was undertaken on liver samples obtained from 93 patients with well characterized NAFLD who underwent percutaneous liver biopsies for their clinical care. We accept that our cohort poses a risk of selection bias. However, our observations are consistent in the meta-analyses with other studies. Therefore, we do not believe any potential selection bias diminishes the validity of our observations.
Ethics oversight	Indiana University Institutional Review Board (protocol numbers: 1506218127, 1011003025R008)

Note that full information on the approval of the study protocol must also be provided in the manuscript.

Field-specific reporting

Please select the one below that is the best fit for your research. If you are not sure, read the appropriate sections before making your selection.

- [x] Life sciences
- [ ] Behavioural & social sciences
- [ ] Ecological, evolutionary & environmental sciences

For a reference copy of the document with all sections, see nature.com/documents/nr-reporting-summary-flat.pdf

Life sciences study design

All studies must disclose on these points even when the disclosure is negative.

Sample size	sample size was based on patient availability and was not statistically determined.
Data exclusions	RNA samples with Agilent Bioanalyzer RIN > 6.0 were advanced for RNA library generation. Only samples with corresponding phenotype information were analyzed.
Replication	findings were reliably validated in 15 other studies. This is presented as a meta-analysis in the manuscript.
Randomization	Samples for RNA-seq were balanced across pools based on distribution of fibrosis stage, NAS score, date of biopsy, age, BMI, date of RNA isolation, RNA quality indicator (RQI), and RNA yield. Clinical groupings were made based on clinical information available and were not randomized.
Blinding	Blinding was not done because these samples were collected over the course of many years of clinical practice by the corresponding author.

Reporting for specific materials, systems and methods

We require information from authors about some types of materials, experimental systems and methods used in many studies. Here, indicate whether each material, system or method listed is relevant to your study. If you are not sure if a list item applies to your research, read the appropriate section before selecting a response.

Materials & experimental systems

n/a	Involved in the study
☒	☐ Antibodies
☒	☐ Eukaryotic cell lines
☒	☐ Palaeontology and archaeology
☒	☐ Animals and other organisms
☒	☐ Clinical data
☒	☐ Dual use research of concern

Methods

n/a	Involved in the study
☒	☐ ChIP-seq
☒	☐ Flow cytometry
☒	☐ MRI-based neuroimaging
